# Mutagenicity testing with transgenic mice. Part II: Comparison with the mouse spot test

**DOI:** 10.1186/1477-3163-4-4

**Published:** 2005-01-27

**Authors:** Ulrich Wahnschaffe, Annette Bitsch, Janet Kielhorn, Inge Mangelsdorf

**Affiliations:** 1 Fraunhofer Institute of Toxicology and Experimental Medicine ITEM, Department of Chemical Risk Assessment, Nikolai-Fuchs-Str. 1, 30625 Hannover, Germany

## Abstract

The mouse spot test, an *in vivo *mutation assay, has been used to assess a number of chemicals. It is at present the only *in vivo *mammalian test system capable of detecting somatic gene mutations according to OECD guidelines (OECD guideline 484). It is however rather insensitive, animal consuming and expensive type of test. More recently several assays using transgenic animals have been developed. From data in the literature, the present study compares the results of *in vivo *testing of over twenty chemicals using the mouse spot test and compares them with results from the two transgenic mouse models with the best data base available, the *lacI *model (commercially available as the Big Blue^® ^mouse), and the *lacZ *model (commercially available as the Muta™ Mouse). There was agreement in the results from the majority of substances. No differences were found in the predictability of the transgenic animal assays and the mouse spot test for carcinogenicity. However, from the limited data available, it seems that the transgenic mouse assay has several advantages over the mouse spot test and may be a suitable test system replacing the mouse spot test for detection of gene but not chromosome mutations *in vivo*.

## Background

This is the second presentation from a project for the International Programme on Chemical Safety (IPCS) evaluating the possible use of transgenic animal mutagenicity assays in toxicity testing and mechanistic research. Part I, preceeding this article, discussed comparison of effects of chemicals using certain transgenic assays with results using the bone marrow micronucleus test.

The assessment of the potential genotoxicity of chemicals *in vivo *is important for both the verification and confirmation of intrinsic mutagenicity and for establishing the mode of action of chemical carcinogens. Although the present trend is to reduce animal testing, *in vitro *data must be confirmed by testing in *in vivo *conditions which take into account whole animal processes like absorption, tissue distribution, metabolism and excretion of the chemical and its metabolites, and overall toxicity [1]. In the mid 1980s, the mouse spot test [2] was suggested as a complementary *in vivo *test to the bacterial mutagenicity assay for detection of mutagenic substances and as a confirmatory test for the identification of carcinogens [3]. The mouse spot test has been used to assess a number of chemicals (see e.g. Additional file 1, see separate file). It is at present the only *in vivo *mammalian test system capable of detecting somatic gene mutations according to OECD guidelines (OECD guideline 484 [4]). However to achieve an acceptable sensitivity, a large number of animals are necessary and it is therefore an expensive type of test and seldom used. More recently assays using transgenic animals have been developed for testing *in vivo *gene mutagenicity. The two transgenic mouse models with the best data base available are the *lacI *model (commercially available as the Big Blue^® ^mouse), and the *lacZ *model (commercially available as the Muta™ Mouse). The present study compares the results of *in vivo *testing of a number of chemicals using the mouse spot test and compares it with results from these two transgenic mouse models.

### Descriptions of test systems

#### Mouse spot test

In the spot test, mouse embryos which are heterozygous for different recessive coat colour genes, are treated *in utero *at gestation day 9–11 with the test substance. The exposed embryo at gestation day 10 contains about 150–200 melanoblasts and each melanoblast has 4 coat colour genes under study [2,5]. The *in utero *exposure may result in an alteration or loss of a specific wild-type allele in a pigment precursor cell resulting in a colour spot in the coat of the adult animal. The frequency of spots is compared with the frequency in sham-exposed controls [2,4].

In the mouse spot test there are 4 possible mechanisms by which the recessive coat-colour alleles can be expressed: 1) gene mutation in the wild-type allele, 2) deficiency (large or small) of a chromosomal segment involving the wild-type allele, 3) nondisjunctional (or other) loss of the chromosome carrying the wild-type allele and 4) somatic recombination (marker gene then homozygous) [5]. Gene mutagenic but also clastogenic effects are detected by this test system.

#### Transgenic mouse models

The transgenic mutation test systems the *lacI *model (Big Blue^® ^mouse), and the *lacZ *model (Muta™ Mouse) are described in detail in the preceding article: Mutagenicity testing with transgenic mice. Part I: Comparison with the mouse bone marrow micronucleus test

## Methods

Data presented in this documentation are the results of an extensive literature research. Concerning data on transgenic mouse assays only primary literature was used. Data on the mouse spot test were extracted from reliable reviews on this item or from primary literature. For all other data informations from secondary literature or data banks were used.

## Results and Discussion

### Comparison of the mouse spot test with transgenic mouse model systems

In the literature search chemicals have been identified that had been tested using the spot test **and **the Muta™ mouse assay (n = 20) or the Big Blue^® ^mouse assay (n = 9) or both transgenic mutation assays (n = 8). The results (including references) are given in Additional file 1.

The results on 15 out of 20 substances (2-acetylaminofluorene, acrylamide, benzo[*a*]pyrene, 1,3-butadiene, cyclophosphamide, ethylmethanesulfonate, *N*-ethyl-*N*-nitrosourea, *N*-methyl-*N*'-nitro-*N*-nitrosoguanidine, *N*-methyl-*N*-nitrosourea, 4-nitroquino-line-1-oxide, *N*-nitrosodiethylamine, *N*-nitrosodimethylamine, procarbazine, 4-acetylaminofluorene and *N*-propyl-*N*-nitrosourea) showed agreement between the Muta™ mouse and the mouse spot test. No agreement was seen with 5 out of 20 substances (4-acetylaminofluorene, 2-amino-3-methylimidazo(4,5-f)quinoline (IQ), hydrazine, mitomycin C, trichloroethylene).

The positive results obtained with the Big Blue^® ^mouse assay agreed with results in the mouse spot test for 7 out of 9 substances (2-acetylaminofluorene, benzo[*a*]pyrene, 1,3-butadiene, cyclophosphamide, *N*-ethyl-*N*-nitrosourea, *N*-methyl-*N*-nitrosourea, *N*-nitrosodimethylamine); one (di-(2-ethylhexyl)phthalate) was negative in both test systems and only one (methyl methanesulfonate) showed no agreement between the two test systems.

With two exceptions, 4-acetylaminofluorene and N-propyl-N-nitrosourea (discussed later), all of the tested substances showed also clearly positive results in *in vitro *gene mutation assays (exception of 1,3-butadiene, negative results) and in the majority of *in vivo *studies on this endpoint. Further they induced carcinogenic effects in long-term studies on mice.

Although no data on carcinogenicity on mice is available on *N*-propyl-*N*-nitrosourea, this substance might also be included in the category mentioned above, since carcinogenic effects were reported in rats [113] and *in vitro *gene mutation assays revealed clearly positive results.

The following substances did not show agreement between results in the mouse spot test and transgenic mouse assays or negative results were reported in both test systems (see Additional file 1). These are therefore discussed in more detail here; for references see Additional file 1.

**Table 1:** Characteristics of the Muta™ mouse assay and the Big Blue^® ^mouse assay for predicting mouse carcinogenicity in comparison with the mouse spot test

Term#	Calculation* for the mouse spot test	Calculation* for Muta™ and/or Big Blue^® ^mouse combined **
Sensitivity	84% (16/18)	79% (15/18)
Specificity	0 (0/0)	0 (0/0)
Positive predictability	100% (16/16)	100% (15/15)
Negative predictability	0 (0/2)	0 (0/3)
Overall accuracy	84% (16/18)	79% (15/18)

# Sensitivity = % of carcinogens with a positive result in the specified test system (STS)

Specificity = % of noncarcinogens with a negative result in the STS

Positive predictivity = % of positive results in the STS that are carcinogens

Negative predictivity = % of negative results in the STS that are noncarcinogens

Overall accuracy = % of chemicals tested where STS results agree with the carcinogenicity results

*: carcinogens with genotoxic and nongenotoxic mechanisms were considered but not substances without data on carcinogenicity; only data on mice were used

**: judged as positive in transgenic assays if positive in one of the two test systems

For methylmethanesulfonate, the weak positive results were judged as positive.

Trichloroethylene was not included in the calculation (inconclusive results in the mouse spot test).

#### 4-Acetylaminofluorene

This substance showed mutagenic activity in the Muta™ mouse assay [19] but negative results in the mouse spot test [12,13]. No data on carcinogenicity are available on 4-acetylaminofluorene. However, data on two *in vitro *test systems indicated gene mutagenic activity supporting results in the transgenic assay [15-18].

#### 2-Amino-3-methylimidazo(4,5-f)quinol (IQ)

IQ is mutagenic in the Muta™ mouse assay [28] but negative results were obtained in the mouse spot test [29]. This negative result in the mouse spot test is in contrast to all other *in vivo *gene mutation assays on rodents and insects which revealed positive results [27]. Furthermore, gene mutagenic activity was detected in *in vitro *test systems and carcinogenic effects were observed in long-term studies on mice [27]. The results in the Muta™ mouse assay are in accordance with these data.

#### Di-(2-ethylhexyl)phthalate

Negative results in the mouse spot test [51] are in agreement with the negative Big Blue^® ^assay [11]. Furthermore no gene mutagenic or questionable activity was reported in *in vitro *tests and in tests on Drosophila. Carcinogenic effects were obtained in studies on mice but nongenotoxic mechanisms are presumed.

#### Hydrazine

This substance induced mutagenic effects in the mouse spot test [72] but negative results were observed in the Muta™ mouse assay [71]. Other *in vivo *as well as *in vitro *test systems revealed gene mutagenic effects [70]. Increased tumor incidences were observed in carcinogenicity studies on mice. Overall, the mouse spot test but not the Muta™ mouse assay reflects data on genotoxicity and carcinogenicity. However, a single exposure was used in the Muta™ mouse assay [71]. Studies on other *in vivo *genotoxicity endpoints have shown generally negative results after single exposure but genotoxic activity after repeated application, for example the mouse bone marrow micronucleus assay was positive [20]. It is possible that positive results may be found using another experimental design in the Muta™ mouse assay e.g. repeated exposure.

#### Methyl methanesulfonate

Only weak mutagenic effects were observed in the Muta™ mouse [19,57,75-77] and negative results in the Big Blue^® ^mouse [63-65,78]. In the mouse spot test this carcinogenic substance is mutagenic [3] as well as in other gene mutation assays *in vitro *and *in vivo *[73,74]. However, there is evidence that the chromosome mutagenic activity is detectable at much lower doses than the gene mutagenic activity. Tinwell et al. [19] have shown in Muta™ mice a weak gene mutagenic effect in the liver but no effect in the bone marrow. The same dose induced in these animals a significant increase in bone marrow micronuclei indicating clear clastogenic activity. However, the transgenic mutation assay is less suitable for detection of these effects [1].

#### Mitomycin C

No mutagenic activity was observed in the Muta™ mouse assay after single application and ambiguous results after repeated exposure [93] but positive results were obtained with the mouse spot test [2,3] and other gene mutation assays *in vitro *and *in vivo *with this carcinogenic substance [90-92]. The reason for this discrepancy is similar to that presumed for methyl methanesulfonate above. Clastogenicity in bone marrow but no gene mutagenic activity in liver and bone marrow has been shown in the same animals in the Muta™ mouse assay combined with a micronucleus assay [93]. However, using another experimental design for detection of gene mutations in the Muta™ mouse assay (dose level up to the MTD, repeated exposure) positive results might be obtained.

#### Trichloroethylene

Also with this carcinogenic substance, no mutagenicity was detected in the Muta™ mouse assay [117], the mouse spot test was positive [3], but this result is possibly related to contaminations with epoxides [116]. Further *in vitro *and *in vivo *assays on gene mutation resulted in weak positive, questionable, or negative effects [116]. Results in chromosome mutation assays are equivocal. However, a further (simple) reason for this discrepancy between the Muta™ mouse assay and the mouse spot test might be that the MTD was not reached in the Muta™ mouse assay presented by Douglas et al. [117].

In general, from the studies on genotoxic carcinogens given above, the results do not seem to give a preference for either the spot test or transgenic mouse model system.

However, considering the mechanisms of action of specific substances there is some evidence, that the mouse spot test detects gene mutations as well as chromosome mutations whereas the transgenic mouse assays are restricted to gene mutations. Evidence for this hypothesis has been shown with the examples methyl methanesulfonate, mitomycin C, and trichloroethylene. In the mouse spot test, there are four possible mechanisms by which the recessive coat-colour alleles can be expressed (see introduction) including gene and chromosome mutations. Although the chromosome mutations have to survive several mitoses to cause the expression of the recessive allele [118], there is evidence that also predominantly clastogenic substances might result in a positive mouse spot test. In contrast, the transgenic mutation assays detected point mutations and maximal small deletions and insertions [1].

### Predictivity of the transgenic animal assays and the mouse spot test for carcinogenicity

The sensitivity, specificity and predictivity of carcinogenicity for the transgenic mouse model (Muta™ mouse assay and the Big Blue^® ^mouse assay combined) and the mouse spot test are documented in Table 1. Data on 18 substances (see Additional file 1) are available on carcinogenicity in mice and mutagenic effects in transgenic mice as well as mutagenic effects in the mouse spot test (trichloroethylene not included because of inconclusive results in the mouse spot test).

Although the data pool is not sufficient for a comprehensive comparison, there is some indication, that no significant differences were detectable between the two test systems.

### Advantages and disadvantages of both test systems

#### Sensitivity of the test system

In comparison to models using endogenous genes like the target genes in the mouse spot test, the spontaneous mutant frequency in transgenic animals is relatively high. This might be due to the fact that bacterial DNA is the target gene (high methylation rate) and/or the transgene is silent and no transcription related repair occurs as in endogenous genes which are more efficiently repaired [1]. However, comparing the number of cells and genes at risk at the time of exposure, the mouse spot test is numerical inferior to the transgenic mouse mutation assays. In the mouse spot test, the exposed embryo at gestation day 10 contains about 150–200 melanoblasts and each melanoblast has 4 coat colour genes under study [2,5]. In the transgenic Big Blue^® ^mouse, for example, 30–40 copies of the target gene (the constructed λLIZα shuttle vector) are integrated on chromosome 4 of **each **cell of the animal [1].

#### Other factors

To achieve an acceptable sensitivity, a large number of animals are necessary in the mouse spot test. Many pregnant dams have to be in one treatment group to get a sufficient number of surviving F1-animals, since the test substance may induce maternal and developmental toxicity. Fahrig [2] suggested that 30–40 pregnant mice are needed per treatment group for evaluation of spots in the progeny. At least 150 F1-mice are recommended for the concurrent vehicle control [5] and at least two dose groups are used (OECD guideline 484 [4]). Therefore, the mouse spot test is an expensive type of *in vivo *test.

In contrast, in transgenic mutation assays ca. 20 animals (3 dose groups and 1 concurrent vehicle control group in laboratories which already established this test system) are recommended per species and gender [119-121].

In the mouse spot test the discrimination between spots of mutagenic and non-mutagenic origin may be problematic [2].

A comparison of both test systems is presented in Table 2.

**Table 2:** Advantages and Disadvantages of mouse spot test compared to the transgenic Big Blue^® ^and Muta™ mouse assays

	**Mouse spot test ^[2-5]^**	**Transgenic mouse mutation assay^[1, 122]^**
Age restriction	Exposure restricted to embryos at gestation day 9–11	Usually less than 3 months
Toxicokinetics and metabolism	Restrictions in toxicokinetics: test substance reaches the fetal melanoblasts after administration to the dams and absorption of the test substance itself or the toxic metabolites via the placenta	No further barrier like the placenta after absorption and distribution
Target tissue	Restricted to melanoblasts	No tissue restriction; analysis of mutagenic potency in different organs
Type of mutation	Detects 1) gene mutation, 2) large or small deletions, 3) loss of the chromosome carrying the wild-type allele and 4) somatic recombination (marker gene then homozygous)	Detects 1) gene mutation, 2) small deletions or insertions
Dependency of effects on application route	Only systemic effects can be detected; no application route specific effects	For different routes systemic as well as local mutagenic effects can be detected
Target gene/cell	4 genes per cell in ca. 200 melanocytes	Ca. 40 (Big Blue) or ca. 80 (Muta™ mouse) copies of the transgene per nucleus of each cell of the organismk
Number of animals	Animal consuming test system	Not more than 5 animals per gender per dose necessary
Specificity of test system	Discrimination between spots of mutagenic and non-mutagenic origin may be problematically	Identifying and isolating mutated genes with a high specificity
Characterisation of mutations by molecular methods	Less suitable for identification of mutations in DNA analysis due to size of the genes	detection of the "molecular signature" of a particular mutagen by DNA sequence analysis with standardized methods
Possibility of parallel investigation of several genetic endpoints	No combination with other genotoxic endpoints possible	The transgenic mouse assay can be combined with other *in vivo *genotoxic endpoints in the same animal: e.g. micronuclei, chromosomal aberration, unsheduled DNA synthesis, sister chromatid exchange
Endogenous versus foreign target gene	The mouse spot test shows an in situ end point (expression of the target genes)	Target genes are integrated parts of foreign DNA and consequently no "normal" mutational target
Costs	Expensive type of *in vivo *test	Less expensive

## Conclusions

Although the mouse spot test is a standard genotoxicity test system according to the OECD guidelines, this system has seldom been used for detection of somatic mutations *in vivo *in the last decades. This is partly due to considerations of cost effectiveness and number of animals needed for testing but also for toxicological considerations. The usefulness of the mouse spot test in toxicology is limited by restrictions in toxicokinetics, sensitivity, target cell/organ, and molecular genetics. From the limited data available, it seems that the transgenic mouse assay has several advantages over the mouse spot test and may be a suitable test system replacing the mouse spot test for detection of gene but not chromosome mutations *in vivo*.

## Author's contributions

UW was the main author. The other authors were involved in the discussions, writing small parts of text and in final preparation of the manuscript.

## Supplementary Material

Additional File 1 Results in the transgenic mouse assay versus mouse spot test Click here for file
