# Supplementary material for: Mutagenicity testing with transgenic mice. Part II: Comparison with the mouse spot test
Source: J Carcinog. 2005 Jan 27;4:4. doi: 10.1186/1477-3163-4-4 (PMC548508; doi:10.1186/1477-3163-4-4)
Supplement: Additional File 1 — Results in the transgenic mouse assay versus mouse spot test [file 1477-3163-4-4-S1.doc]

**Additional file 1**

Results in the transgenic mouse assay versus mouse spot test

| Substance^a^ | **Results in carcinogenicity studies on mice [IARC evaluation]** | **Results in transgenic assays** | | **Results of mouse spot test** | **Agreement   mouse spot test  with** | | Further gene mutation assays | |
| --- | --- | --- | --- | --- | --- | --- | --- | --- |
|  |  | **Muta mouse** | **BB mouse** |  | **Muta mouse** | **BB mouse** | ***in vitro*** | ***in vivo*** |
| 2-Acetylamino-fluorene ^6,78^ | Positive  [none] | +^9^ | +^6,10,11^ | **+**^12,13,14^ | yes | yes | **++** | **±** (Drosophila, SLRL) |
| 4-Acetylamino-fluorene ^15-18^ | No data  [none] | +^19^ | nd | **-**^12,13^ | no | na | **++** | nd |
| Acrylamide^20-22^ | Positive  [2A] | +^23-25^ | nd | **+**^26^ | yes | na | **++** | + (specific locus, mouse) + (host mediated) + (Drosophila, SLRL) + (Drosophila, somat.) |
| 2-Amino-3-methylimidazo(4,5-f)quinol (IQ)^27^ | Positive  [2A] | +^28^ | nd | **-**^29^ | no | na | **++** | + (HPRT, rat) + (host mediated) + (Drosophila, SLRL) + (Drosophila, somat.) |
| Benzo[*a*]pyrene^30,31^ | Positive  [2A] | + ^32,33^ | + ^34-38^ | **+**^3^ | yes | yes | **++** | + (Drosophila, somat.) - (Drosophila, SLRL) |
| 1,3-Butadiene^39^ | Positive  [2A] | +^40^ | + ^40-42^ | **+**^43^ | yes | yes | **-** | + (HPRT, human & mouse)  + (Drosophila, somat.) - (Drosophila, SLRL) |
| Substance^a^ | **Results in carcinogenicity studies on mice [IARC evaluation]** | **Results in transgenic assays** | | **Results of mouse spot test** | **Agreement   mouse spot test  with** | | Further gene mutation assays | |
|  |  | **Muta mouse** | **BB mouse** |  | **Muta mouse** | **BB mouse** | ***in vitro*** | ***in vivo*** |
| Cyclophos-phamide^44,45^ | Positive  [1] | +^23,24^ | +^34, 46-48^ | **+**^3,49^ | yes | yes | **++** | + (host mediated) + (Drosophila, somat.) + (Drosophila, SLRL) |
| Di-(2-ethylhexyl) phthalate^50^ | Positive but nongenotoxic | nd | -^11^ | **-**^51^ | na | yes | **--** | **±** (Drosophila, somat.) - (Drosophila, SLRL) |
| Ethylmethane-sulfonate^52-55^ | Positive  [2B] | +^56-58^ | nd | **+**^2,3,14^ | yes | na | **++** | + (specific locus test, mouse) + (host mediated) + (Drosophila, SLRL) |
| *N*-Ethyl-*N*-nitrosourea^59,60^ | Positive  [2A] | +^24,40,56, 61,62^ | +^63-69^ | **+**^3,14,49^ | yes | yes | **++** | + (specific locus test) + (HPRT, mouse) + (Drosophila, SLRL) |
| Hydrazine &  hydrazine sulfate^70^ | Positive  [2B] | -^71^ | nd | **+**^72^ | no | na | **++** | + (host mediated) + (Drosophila, SLRL) + (Drosophila, somat.) |
| Methyl methane-sulfonate^73,74^ | Positive  [2B] | (+)^19,57,75-77^ | -^63-65,78^ | **+**^3^ | (yes) | no | **++** | **±** (specific locus test) + (HPRT, rat) + (host mediated) + (Drosophila, somat.) + (Drosophila, SLRL) |
| *N*-Methyl-*N*'-nitro-*N*-nitroso-guanidine^79-81^ | Positive  [2A] | +^23,82,83^ | nd | **+**^2^ | yes | na | **++** | + (host mediated) + (Drosophila, somat.) + (Drosophila, SLRL) |
| *N*-Methyl-*N*-nitrosourea^84,85^ | Positive  [2A] | +^86^ | +^87-89^ | **+**^3^ | yes | yes | **++** | + (host mediated) + (Drosophila, SLRL) |
| Substance^a^ | **Results in carcinogenicity studies on mice [IARC evaluation]** | **Results in transgenic assays** | | **Results of mouse spot test** | **Agreement   mouse spot test  with** | | Further gene mutation assays^a^ | |
|  |  | **Muta mouse** | **BB mouse** |  | **Muta mouse** | **BB mouse** | ***in vitro*** | ***in vivo*** |
| Mitomycin C^90-92^ | Positive  [2B] | -^93^ | nd | **+**^2,3^ | no | na | **++** | + (specific locus test) + (host mediated) + (Drosophila, SLRL) |
| 4-Nitroquinoline-1-oxide^86,94,95^ | Positive  [no evaluation] | +^86,96^ | nd | **+**^2,49^ | yes | na | **++** | + (host mediated) |
| *N*-Nitrosodi-ethylamine^97-99^ | Positive  [2A] | +^57,58,100,101^ | nd | **+**^3^ | yes | na | **++** | - (specific locus test) + (host mediated) + (Drosophila, SLRL) |
| *N*-Nitrosodi-methylamine^102, 103^ | Positive  [2A] | +^104^ | +^78,87,105-107^ | **+**^2^ | yes | yes | **++** | + (Drosophila, SLRL) |
| Procarbazine^108,109^ | Positive  [2A] | +^23,24,110,111^ | nd | +^3,49,112^ | yes | na | **++** | + (specific locus test) + (host mediated) + (Drosophila, somatic) + (Drosophila, SLRL) |
| *N*-Propyl-*N*-nitrosourea ^113,114^ | No data  [no evaluation] | +^115^ | nd | **+**^3^ | yes | na | **++** | nd |
| Trichloroethylene^116^ | Positive  [2A] | -^117^ | nd | **±**^3,116^ | inconclusive | na | **(+)** | - (host mediated) |

a: data on carcinogenicity and data in the column “further gene mutation assays” are taken from sources (mostly secondary literature) cited in this column.

BB: Big Blue®; - : negative study results; +: positive (for transgenic mouse assays: at least one examined organ shows an increased mutation frequency); ++ majority of results are positive concerning two or more endpoints in in vitro studies; (+): study result weakly positive; **±**: inconclusive result; nd: no data available; na: not applicable because transgenic assay in this mouse line not done.
